# RNA-binding proteins: a novel target for modulating glucose and lipid metabolism

**DOI:** 10.1038/s41392-025-02236-5

**Published:** 2025-05-02

**Authors:** Yongjian Hu, Qian Sun

**Affiliations:** 1 Department of Biotherapy, Cancer Center and State Key Laboratory of Biotherapy, West China Hospital, Sichuan University, Chengdu, 610041 China; 2 West China Medical Publishers, West China Hospital, Sichuan University, Chengdu, 610041 China

**Keywords:** Endocrine system and metabolic diseases, Endocrine system and metabolic diseases

In a recent study published in *Science*, Chen and colleagues unveiled the mechanism by which hepatic alkylation repair homolog protein 5 (ALKBH5) regulates the glucagon receptor (GCGR) and mechanistic target of rapamycin complex 1 (mTORC1) signaling pathways via two independent mechanisms, thereby integrating the modulation of glucose and lipid metabolism homeostasis.^1^ This research explores the regulation of metabolic homeostasis and the pathogenesis of metabolic diseases from the perspective of RNA-binding proteins, offering new drug targets for alleviating metabolic-associated fatty liver disease (MAFLD) and metabolic disorders.

Blood glucose and lipid levels are maintained by a complex regulatory system. The liver responds to glucagon and insulin to regulate blood glucose levels through glycogen synthesis, glycogenolysis, and gluconeogenesis.^2^ However, in obesity and type 2 diabetes, these regulatory pathways are dysregulated, leading to excessive hepatic gluconeogenesis, elevated fasting blood glucose, increased lipid synthesis, and metabolic disorders such as hyperglycemia, hyperlipidemia, and MAFLD.^2^ Deciphering the mechanisms of hepatic metabolic dysregulation is important for the treatment of obesity, type 2 diabetes, and MAFLD.

Focusing on RNA-binding proteins (RBPs), Chen’s team investigated their regulation of glucose and lipid metabolic homeostasis and their role in metabolic diseases. The team first conducted quantitative proteomic analysis in the liver of db/db mice, a diabetic mouse model, and identified seven significantly upregulated RBPs associated with translation, mitochondrial gene expression, and ribosome biogenesis. By integrating proteomics and phosphoproteomic analysis, ALKBH5 was identified as a key player. ALKBH5 is an mRNA m^6^A demethylase containing the protein kinase A (PKA) recognition motif (RRXS/T), which can be phosphorylated by the glucagon-cAMP-PKA signaling cascade. Subsequent studies in db/db mice, high-fat diet-fed mice, and liver samples from diabetic patients consistently revealed aberrantly increased ALKBH5 expression.

In order to elucidate the molecular basis of glucagon signaling regulation, the team combined AlphaFold structure predictions with in vitro kinase assays to identify Ser^362^ as a functional phosphorylation site of ALKBH5 in response to glucagon-PKA signaling. This phosphorylation of ALKBH5 promotes its translocation from the nucleus to the cytoplasm, where it binds to the m^6^A-modified region of *Gcgr* mRNA, leading to m^6^A demethylation and stabilization of *Gcgr* mRNA, thereby sustaining the GCGR signaling pathway. Hepatocyte-specific *Alkbh5*-knockout (*Alkbh5*-HKO) mice or mice carrying the S362A point mutation exhibited significantly reduced GCGR expression and downstream signaling activation. These genetic modifications resulted in reduced blood glucose levels and conferred resistance to high-fat diet-induced metabolic dysfunction characterized by hyperglycemia and glucose intolerance. Notably, rescue experiments revealed that the demethylase-inactive point mutation (H205A) could not restore GCGR signaling or blood glucose levels in *Alkbh5*-HKO mice. These findings underscore the critical roles of Ser^362^ phosphorylation and ALKBH5 demethylase activity in maintaining GCGR signaling and glucose metabolism homeostasis.

**Fig. 1:**
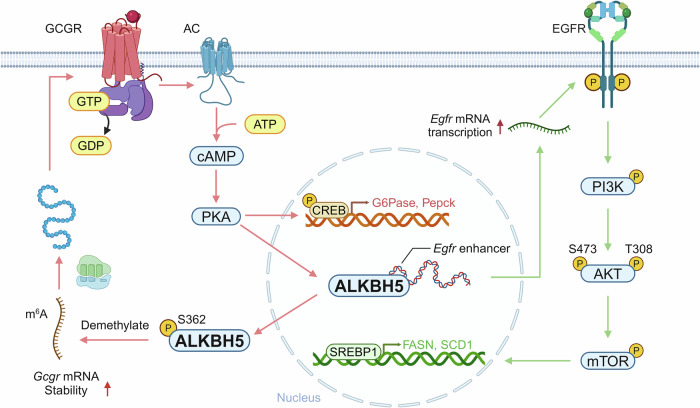
Molecular Mechanisms Underlying ALKBH5-Mediated Regulation of Hepatic Glucose and Lipid Metabolism. ALKBH5 regulates GCGR mRNA stability through its demethylase activity, thereby modulating glucose metabolism signaling pathways. In contrast, ALKBH5 regulates lipid metabolism by enhancing EGFR expression and subsequently modulating the downstream PI3K-mTOR signaling pathway. Created with BioRender.com

Given the central role of the liver in lipid metabolism, the authors further investigated the impact of ALKBH5 on hepatic lipid homeostasis. They found that in *Alkbh5*-HKO mice, the phosphatidylinositol 3-kinase (PI3K)-AKT-mTORC1-sterol regulatory element–binding protein 1 (SREBP1) signaling pathway was significantly downregulated. Additionally, the expression of lipid synthesis-related enzymes (SCD1 and FASN) and the free fatty acid uptake-related protein CD36 were also decreased. Under high-fat diet condition, de novo lipogenesis was significantly suppressed in *Alkbh5*-HKO mice, while β-oxidation remained unaffected. However, in *Alkbh5*-HKO mice, the expression and activation of the insulin receptor (INSR), a classical PI3K regulator, remained unchanged. By integrating RNA sequencing data and analyzing receptor tyrosine kinases that can activate PI3K, the authors identified that epidermal growth factor receptor (EGFR) expression and phosphorylation were reduced in the liver of *Alkbh5*-HKO mice. Restoration of EGFR reversed the downregulation of the PI3K-mTORC1 signaling pathway, thereby alleviating metabolic-associated fatty liver disease and hyperlipidemia phenotypes. Further mechanistic studies revealed that the regulation of EGFR expression by ALKBH5 was demethylase independent and instead depended on two conserved loops (Q145-G152 and C231-E242). These loops specifically bound to the GC-enriched sequence within the *Egfr* enhancer, driving *Egfr* transcription and subsequently upregulating the EGFR-mTORC1 signaling pathway. Interestingly, reintroducing GCGR in the livers of *Alkbh5*-HKO mice reversed the hyperglycemia phenotype but had no effect on lipid metabolism. Conversely, restoration of EGFR ameliorated the metabolic-associated fatty liver disease and hyperlipidemia phenotypes without affecting blood glucose levels. This demonstrates that ALKBH5 regulates the GCGR and EGFR-mTORC1 signaling cascades via distinct molecular processes, coordinating systemic glucose and lipid balance (Fig. 1). While *Alkbh5*-HKO mice exhibited no changes in white adipose lipolysis signaling, the detectable expression of ALKBH5 in metabolic tissues, including skeletal muscle, inguinal/epididymal white adipose tissue (iWAT/eWAT), and interscapular brown adipose tissue (iBAT), suggests potential mechanisms or unexplored functions requiring further investigation.

Using AAV-mediated shRNA and the clinically applicable GalNAc-siRNA technology, the specific knockdown of hepatic *Alkbh5* reversed hyperglycemia, hyperlipidemia, and the MAFLD phenotype in the db/db mouse model. This highlights the therapeutic promise of targeting ALKBH5. Currently, several drugs targeting GCGR to simultaneously reduce blood glucose and lipid accumulation have entered clinical development, demonstrating potential for treating obesity, metabolic disorders, and type 2 diabetes.^3^ For example, retatrutide, a GCGR/glucose-dependent insulinotropic peptide receptor (GIPR)/glucagon-like peptide-1 receptor (GLP-1R) triple agonist developed by Eli Lilly, has entered Phase III clinical trials. The GCGR/GLP-1R dual agonists mazdutide and survodutide have also advanced to Phase III clinical trial, while efinopegdutide has progressed to Phase II clinical development.^4^ Additionally, a recent study published in *Nature* reported a strong correlation between constitutive GCGR activation and elevated blood glucose levels in birds.^5^ This finding not only underscores the functional diversity of GCGR across species but also offers new insights into its role in human metabolic diseases. Unlike multi-target drug strategies, this study demonstrates that a single target - ALKBH5 - can coordinately regulate both glucose and lipid metabolism through distinct mechanisms: (1) m^6^A demethylase activity-dependent modulation of the GCGR pathway for glucose homeostasis, and (2) demethylase independent regulation of EGFR-mTORC1 axis for lipid homeostasis. These dual metabolic improvements, achieved through a single molecular target, highlight ALKBH5’s distinctive translational value for treating metabolic disorders, warranting further preclinical validation and mechanistic studies to facilitate clinical development.
